# SaurKshetra: A curated dataset and ML-based classification for solar energy site selection

**DOI:** 10.1016/j.dib.2026.112906

**Published:** 2026-06-04

**Authors:** Yash Shivgan, Chandan Bhirud, Anshul Jamgade, Atharva Kulkarni, Rutuja Patil, Gagandeep Kaur, David Bassir, Ghanshyam G. Tejani

**Affiliations:** a Vishwakarma Institute of Information Technology, Kondhwa Budruk, Pune, India; b Department of Computer Engineering, MIT, Academy of Engineering, Alandi, Pune, India; c Department of Computer Engineering, MPSTME, SVKM’s NMIMS University, Maharashtra, India; d Smart Structural Health Monitoring and Control Laboratory, DGUT-CNAM, Dongguan University of Technology, China; e ENS -Paris-Saclay University, Centre Borelli, UMR CNRS 9010, 91190, Gif-sur-Yvette, France; f Department of Research Analytics, Saveetha Dental College and Hospitals, Saveetha Institute of Medical and Technical Sciences, Saveetha University, Chennai 600077, India; g Jadara Research Center, Jadara University, Irbid, 21110, Jordan

**Keywords:** Solar energy dataset, Site selection, Renewable energy, Machine learning, Geospatial analysis

## Abstract

The global demand for clean energy is on the rise and the best solar power sites among various regions need to be identified. India has the potential to become an important source for solar energy due to its vast and diverse geographical area, however, site selection based on accurate data is necessary for the effective utilization. The present research work has provided a geospatial dataset of monthly environmental variables—such as global horizontal irradiance, clear-sky irradiance, surface albedo, cloud cover, temperature at 2 m, dew point, and precipitation—acquired at 371 sites in India from January 2013 to December 2022. The data is divided into five regional zones—North, East, West, Central, and South— and is available in XLSX format for ease of mapping and analytical workflows. A weighted scoring method is used for the selected variables to sort the sunniest spots into five categories of suitability ranging from Excellent to Unsuitable. K-means clustering helps in categorizing locations based on their solar power potential, whereas a Random Forest model fine-tunes the classification into the designated suitability classes. The spatial maps and rankings generated, thus, provide a detailed summary of the solar energy potential throughout India. Besides, all datasets and classification outputs have been made available for free on Mendeley Data (DOI: *10.17632/hkcyvhvmwp.5*) as a ready-to-use resource for researchers, planners, and policymakers.

Specifications Table

**Table:** 

Subject	Computer Sciences
Specific subject area	Geospatial Analysis and Machine Learning for Solar Site Suitability Mapping
Type of data	XLSX files (raw and processed)
Data collection	Environmental data records on a monthly basis for the period of January 2013 to December 2022 were obtained through the NASA POWER Data Access Viewer API using a 1° × 1° grid. For the analysis, the Surface Downward Irradiance (All-Sky and Clear-Sky), Surface Albedo, Cloud Amount, Temperature at 2 m, Dew/Frost Point at 2 m, and Precipitation were among the seven significant variables that were selected to derive the data for 371 unique site locations distributed in the five different Indian zones. The data were then saved in XLSX format for further use in ML and GIS workflows. The individual grid cells were combined into five regions, which were North, East, West, Central, and South.
Data source location	The dataset is obtained from the NASA POWER Data Access Viewer, provided by NASA Langley Research Centre, ensuring globally recognized scientific accuracy and reliability.
Data accessibility	Repository name: SaurKshetra: A Dataset for Solar Farms Potential Site Mapping using Suitability parameters in India Data identification number: 10.17632/hkcyvhvmwp.5 Direct URL to data: https://data.mendeley.com/datasets/hkcyvhvmwp/5 Instructions: The data is publicly available and can be accessed anonymously using the provided URL0
Related research article	*None*

## 1 Value of the Data

• The dataset is distributed as a single consolidated file Final_Dataset.xlsx, which contains records for 371 georeferenced sites across India covering the period 2013–2022 [1]. This dataset compiles monthly measurements from January 2013 – December 2022 for seven key environmental parameters—solar irradiance, cloud cover, albedo, temperature, dew point, and precipitation—across 371 locations. Because the data cover all seasons and zones, the dataset enables detailed regional and seasonal comparisons of solar potential, as in recent solar resource atlases that present monthly-average maps for each month. The main columns in Final_Dataset.xlsx are LAT, LON, ZONE, YEAR, GHI, CLEAR_GHI, ALBEDO, CLOUD_COVER, TEMP_2M, DEW_POINT, PRECIPITATION, and SUITABILITY.
• The dataset can support machine learning workflows by offering clean, pre-processed inputs for training models that classify or rank locations based on solar suitability. Researchers can apply clustering or supervised algorithms without additional preprocessing steps, making the dataset suitable for experimentation, benchmarking, and reproducible ML pipelines [2]. This curated file harmonizes grid-resolution differences and aggregates monthly values, reducing the preprocessing burden compared with directly downloading raw NASA POWER outputs.
• The classification system integrated into the dataset assigns each location a suitability level from “Excellent” to “Poor,” based on weighted environmental parameters [3]. This structured ranking facilitates practical decision-making by solar developers, energy consultants and policymakers focused on infrastructure investment and grid planning.
• Geospatial visualizations of this dataset, which include solar potential maps and region-wise suitability overlays, provide a user-friendly way to explore the solar energy potential in India. These visual tools facilitate the communication of technical results to the stakeholders such as government, utility companies, and private developers [4].
• The dataset's open-access nature, delivered in XLSX format with standardized labels and zone categories, guarantees its usability across the board [5]. Furthermore, it can be effortlessly incorporated into GIS platforms or applied to analogous classification tasks in different nations, thus increasing its importance for global comparative studies in renewable energy planning.

## 2 Background

**Fig. 1:**
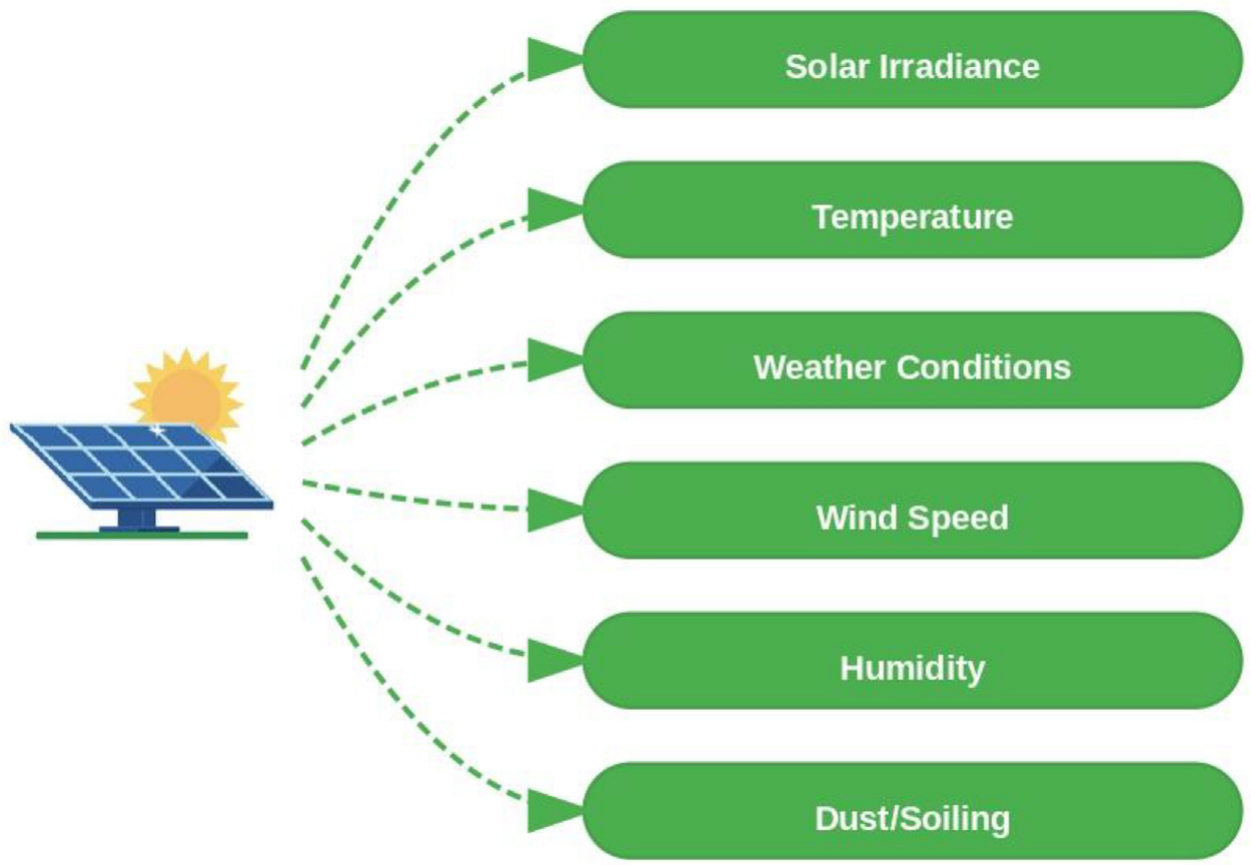
Environmental factors affecting solar energy generation.

The modern world is increasingly adopting renewable energy options with solar power coming out as the main contender against conventional energy. What makes solar energy unique is that its efficiency is influenced by various natural factors like the amount of sunlight and temperature, as well as the amount of clouds and the light reflected by the Earth's surface. Selection of the location for the installation of solar energy systems is very important as these factors vary a lot depending on the place. The environmental factors impacting power production are shown in Fig. 1.

The precise calculation of these factors and regular data monitoring lead to improving the forecasts of energy production capacity depending on location [6]. The market for solar power in India needs accurate distribution of resources as it carries a lot of light in power generation.

AI and ML applications for making real-world problem solutions have undergone major development over the past few years especially in the area of energy [6]. Modern technologies enable advanced pattern detection and performance prediction and location classification for solar installations which surpasses previous methods [2]. Accessing dependable data from NASA POWER enables improved solar panel placement decisions and supports the development of sustainable energy systems through effective use of reanalysis datasets, machine-learning forecasting, and geospatial analysis to identify high-potential sites, quantify uncertainty, and inform planning and policy.

This study leverages those advances to provide a reproducible, data-driven framework for solar resource assessment and site selection in data-scarce regions.

## 3 Data Description

**Fig. 2:**
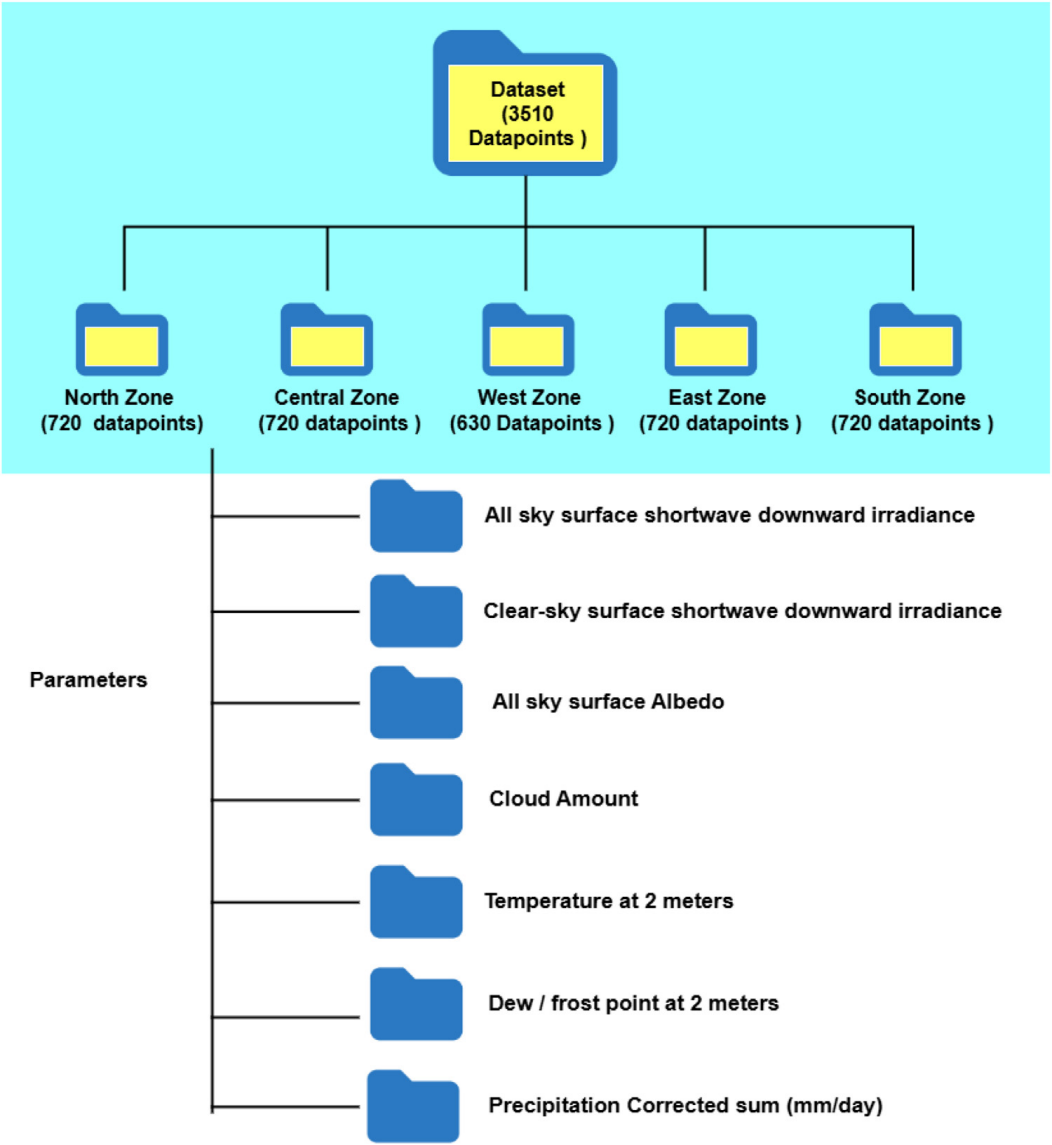
Organization of the dataset: each column in Fig. 2 corresponds to a column in Final_Dataset.xlsx (with zone and parameter groupings as indicated).

The Surface Irradiance and Climate Dataset contain monthly climate and solar-radiation measurements for January 2013 – December 2022 at 371 georeferenced sites across India (latitudes 10.5°–36.5° N, longitudes 68.5°–94.5° E). For each site we recorded seven variables: All-Sky Surface Shortwave Downward Irradiance (GHI), Clear-Sky Surface Shortwave Downward Irradiance (Clear_GHI), Surface Albedo, Cloud Amount, Temperature at 2 m (TEMP_2M), Dew/Frost Point at 2 m (DEW_POINT), and Precipitation (Corrected Sum).Sites are grouped into five geographic zones—North, East, West, Central, and South—to capture regional environmental variability see Fig. 2.

All processed data are provided as a single consolidated file (Final_Dataset.xlsx) that integrates records from all five geographic zones (North, East, West, Central, and South). Each row in Final_Dataset.xlsx represents a single site–year record, defined by a unique latitude–longitude coordinate (LAT, LON), its geographic zone, the corresponding year (YEAR), and the aggregated environmental variables. The main columns include: LAT (latitude), LON (longitude), ZONE (regional classification: North, East, West, Central, South), YEAR, and seven environmental variables retrieved from NASA POWER: GHI, CLEAR_GHI, ALBEDO, CLOUD_COVER, TEMP_2M, DEW_POINT, and PRECIPITATION. The dataset consists of 3510 records in total, which are from 371 unique spatial locations, indicating that monthly NASA POWER observations for the period 2013–2022 were first retrieved and then aggregated into annual values for each site–year. 3510 record 371 values produced after the final dataset contains 3,510 rows after standard preprocessing and consistency checks during dataset preparation. This ensures that the released dataset contains only valid and consistent records suitable for downstream analysis. Irradiance, albedo, cloud amount, temperature and dew point were aggregated using annual mean values, while precipitation was aggregated using annual sum values. The spatial coverage of the different zones has led to an uneven distribution of the data points among the zones, as illustrated in Fig. 2. The use of this single file format means that there are no longer any zone-specific folders, access to the data is made easy, and integration into GIS and machine learning workflows has been made smooth. By making a single standardized XLSX file that contains all variables and regions, the dataset does not only allow for clear-cut analysis but also helps in the comparing and studying of large-scale research without being ambiguous.

## 4 Experimental Design, Materials and Methods

### 4.1 Experimental design

**Fig. 3:**
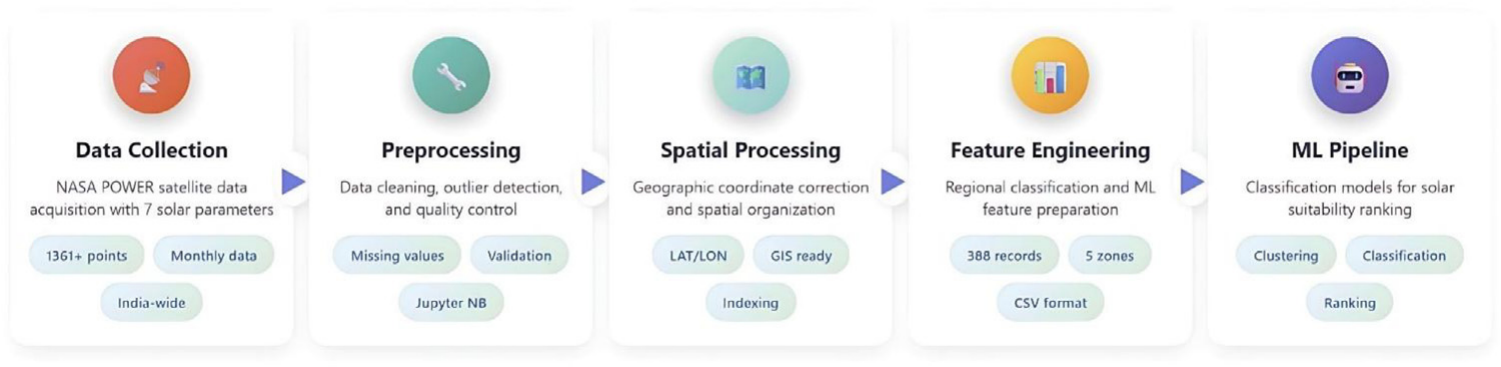
Workflow for satellite-based suitability assessment.

As shown in Fig. 3, a sequence of stages started with the extraction of data from different sources to forming different presentations of the conclusions. The data extraction was done by using the NASA POWER Data Access Viewer API [7]. The first data capture activity included 3510 data points, each of which represented a specific latitude–longitude location with a set of the monthly values of the seven environment parameters that had been aggregated over the period of ten years (2013–2022). The preprocessing of latitude and longitude was a must because the NASA POWER dataset applied different spatial grid resolutions for different parameters. In the first round of data acquisition, four parameters' longitude values were taken at 0.5° intervals (for instance, 79.5°, 80.0°, 80.5°), while the rest of the three parameters were taken at 0.625° intervals. Such variations resulted in the parameters having different latitude–longitude coordinates that needed to be aligned before merging the datasets. This analysis of the dataset indicated that we had to process it via Jupyter Notebook code. The processing of data resulted in the identification of 371 specific rows which corresponded to the five major zones of India: North, East, West, Central, and South. The final data set was thoroughly sorted and put into specific folders based on their classification to ensure maximum accessibility.

We retrieved data from the NASA POWER Data Access Viewer API for the period Jan 2013–Dec 2022. The query covered a bounding box over India (latitudes 10.5°–36.5° N, longitudes 68.5°–94.5° E) at 1° × 1° resolution. We requested monthly (not climatology) data for the following parameters (with their NASA API variable names):

1 ALLSKY_SFC_SW_DWN (All Sky Surface Downward Irradiance)
2 CLRSKY_SFC_SW_DWN (Clear Sky Shortwave Downward Irradiance)
3 ALLSKY_SRF_ALB (All Sky Surface Albedo)
4 CLD_AMT (Daylight Cloud Amount)
5 T2M (Air Temperature at 2 m)
6 T2MDEW (Dew/Frost Point at 2 m)
7 PRECTOTCORR_SUM (Precipitation Corrected Sum).

The data were downloaded in April 2025 via the POWER API (see repository code). Any missing or flagged values in the raw data were handled by removing those site–year rows (no imputation was applied).

The Solar dataset encompasses various solar irradiance measurements and environmental parameters covering ten years of monthly data from NASA POWER Data Access Viewer. The dataset highlights the major climate and geographic factors that determine the solar energy location evaluation thus providing an extensive understanding of solar power potentials. The data collection process involved getting the information of every month of the whole year in order to have a full understanding of solar power plants potential. During pre-processing a Python script was used to create the standardized data formats that the analysis requires for uniformity.

The total number of observations for all seven environmental metrics in each zone is presented in Table 1. The South zone provides the highest data share (25.8%), and the East and North zones follow, thus guaranteeing an even regional representation.

**Table 1:** Breakdown of row distribution of the dataset.

Zone	Observations per Parameter *(All 7 Parameters)*	Total Observations *(× 7 Parameters)*	% of Total Dataset
North	72	504	19.40%
East	71	497	19.13%
Central	72	504	19.40%
West	63	441	16.98%
South	93	644	24.79%
All Zones	***371* (sum per-parameter)**	**2,597**	**100%**

The final dataset contains 3510 rows, where each row represents a site–year record corresponding to a specific latitude–longitude location and year within the study period (2013–2022). Each row includes aggregated values of seven environmental parameters derived from monthly NASA POWER observations.

In contrast, the 2597 observations reported in Table 1 refer to parameter-level observations across all locations for a single aggregated year. Thus, 3510 represents site–year records, while 2597 represents parameter observations, reflecting different levels of aggregation rather than conflicting counts

Data acquisition from the source marked the beginning of the process. The data underwent a quality assessment followed by standardization before entering the final dataset

### 4.2 Methods

A Jupyter Notebook-based Python script conducted analysis on the dataset to evaluate multiple locations for solar power plant installations. The methodology combines statistical analysis with dimensionality reduction together with clustering and classification and machine learning techniques Fig. 2, Fig. 3 to evaluate the selected parameters thoroughly.

#### 4.2.1 Data loading and preprocessing

The Excel dataset comprises geographical coordinates and seven solar-relevant environmental parameters which include latitude and longitude [8], which has been extracted and pre-processed manually.

#### 4.2.2 Advanced analytical techniques

**Fig. 4:**
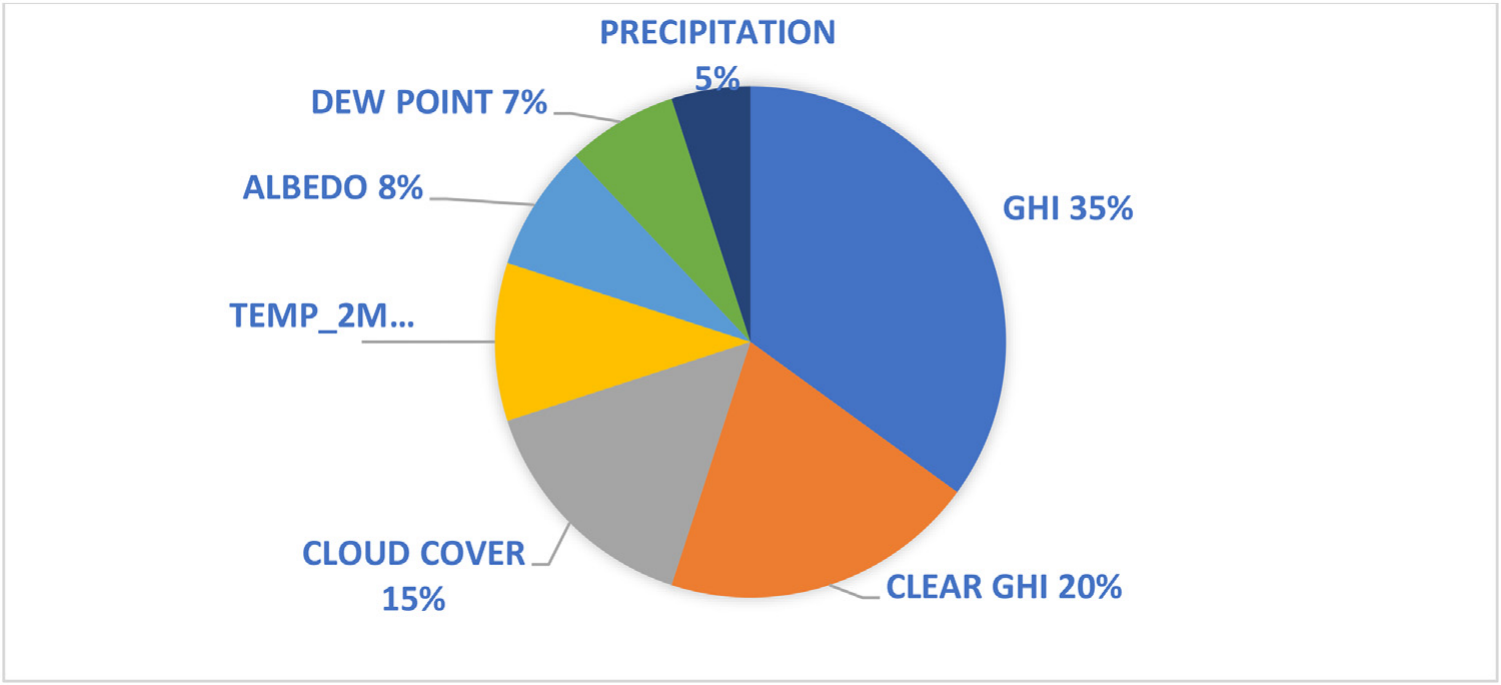
Weightage according to parameter relevance.

Principal Component Analysis (PCA) served to reduce dimensionality while maintaining vital variance in the data. K-Means Clustering (k = 3) [9] was applied to group sites based on their solar irradiance profiles using the features GHI, CLEAR_GHI, CLOUD_COVER, and TEMP_2M. Feature scaling was performed using StandardScaler before clustering to ensure equal weighting of variables Fig. 4. The two-sample t-tests as hypothesis testing methods determined if temperature differences between high and low solar irradiance regions were statistically important.

Fig. 4 illustrates the relative weight assigned to each environmental parameter in the solar site suitability scoring framework. These weights represent the contribution of individual variables to the overall suitability score used to evaluate whether a given latitude–longitude location is favourable for solar power plant deployment. Global Horizontal Irradiance (GHI) is the most significant factor in the solar energy availability calculation, thus it is assigned a weight of 35%. Clear-sky irradiance (20%) and cloud cover (15%) are also heavily weighted since they determine the amount of effective solar radiation that the surface receives directly. Temperature at 2 m (10%) and surface albedo (8%) represent the effects of thermal efficiency and surface reflectivity, while dew point (7%) and precipitation (5%) receive lower weights due to their indirect but still significant influence on long-term operational performance. The distribution of weights is founded on recognized solar energy literature and expert opinion and is meant for suitability ranking purposes only, not for direct energy prediction.

#### 4.2.3 Exploratory data analysis (EDA)

**Fig. 5:**
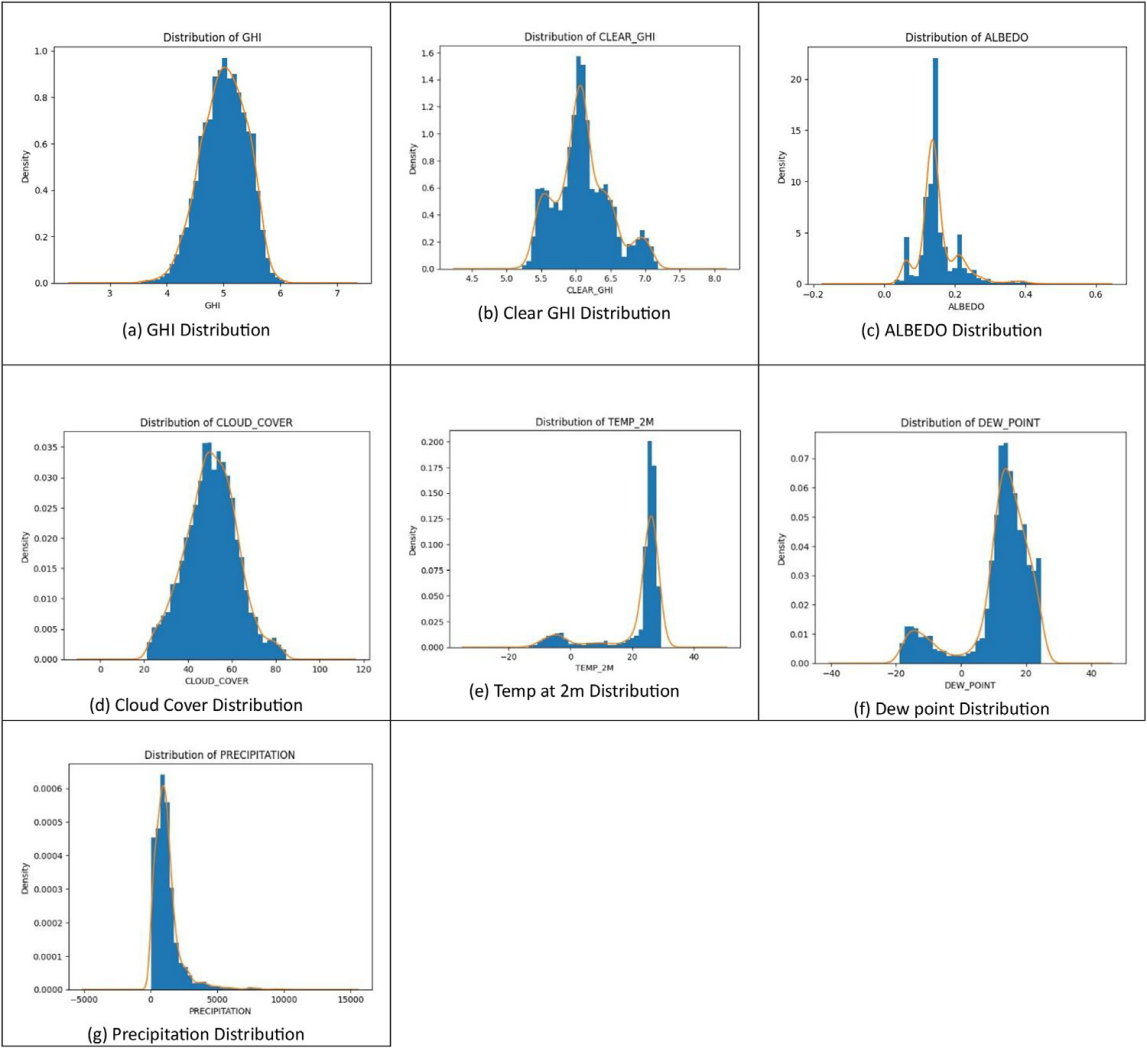
Distribution of meteorological parameters.

After applying clustering and dimensionality reduction, exploratory data analysis was conducted to reveal the statistical features of each variable. For every parameter, descriptive statistics, namely mean, standard deviation, minimum, maximum, and quartile values, were calculated. To visualize the data distributions and to spot possible outliers, boxplots along with histograms and kernel density estimates (KDEs) superimposed were produced, and they are presented in Fig. 5. Besides, Variance Inflation Factor (VIF) analysis was executed to check multicollinearity among features and guarantee the robustness of the analysis.

#### 4.2.4 Suitability classification

A weighted suitability scoring framework was used to classify locations into five solar suitability categories: Excellent, Good, Moderate, Poor, and Unsuitable Fig. 7. The weights given to each environmental factor show their relative importance for the efficiency of solar power generation and were set in advance according to the knowledge and results from the literature [10]. The distributional characteristics of the parameters as shown by histograms and kernel density estimates (Fig. 5) were only for exploratory data analysis to recognize the variability, central tendencies, and possible outliers throughout the dataset. The visualizations guided the qualitative evaluation of parameter behavior but were not straightaway utilized to calculate or derive the weights. Instead, the weight values shown in Fig. 4 represent literature-informed importance rankings of irradiance, atmospheric, and surface-related factors in solar site suitability evaluation.

The overall suitability score was computed as a weighted sum of normalized environmental variables:

Score = 0.35 · norm(GHI) + 0.20 · norm(CLEAR_GHI) + 0.08 · norm(ALBEDO)

+ 0.15 · (1 − norm(CLOUD_COVER))

+ 0.10 · (1 − norm(TEMP_2M))

+ 0.07 · (1 − norm(DEW_POINT))

+ 0.05 · (1 − norm(PRECIPITATION))

where norm(x) denotes min–max normalization applied to each variable so that values fall within the range [0,1]. Variables that negatively affect solar suitability—cloud cover, temperature, dew point, and precipitation—were inverted using 1 − norm(x) so that higher normalized values consistently represent more favorable solar conditions.

Based on the resulting score distribution, each location was categorized using the following thresholds:

Excellent: Score ≥ 0.80
Good: 0.65 ≤ Score < 0.80
Moderate: 0.50 ≤ Score < 0.65
Poor: 0.35 ≤ Score < 0.50
Unsuitable: Score < 0.35

These thresholds ensure that the classification aligns with the weight distribution shown in Fig. 4 and allow other researchers to reproduce the suitability classes directly from the environmental variables. All environmental variables were normalized using min–max scaling prior to computing the suitability score so that differences in units and value ranges did not bias the weighted scoring framework.

#### 4.2.5 Machine learning modelling

For automated classification, a Random Forest classifier [11] was trained using the processed environmental dataset. The input features consisted of the seven environmental variables, while the target variable corresponded to the suitability class derived from the weighted scoring framework.

Before modelling, all numerical features were standardized using StandardScaler to ensure consistent scaling across variables.

The dataset was split into training (80%) and testing (20%) subsets using train_test_split(random_state=42).

The Random Forest classifier was implemented using scikit-learn’s RandomForestClassifier with the following parameters:

• n_estimators = 100
• max_depth = None
• min_samples_leaf = 1
• random_state = 42

Model performance was evaluated using accuracy, precision, recall, and F1-score on the test dataset.

To interpret the model predictions, SHAP (SHapley Additive exPlanations) values were computed using the Python shap library [12]. SHAP analysis quantifies the contribution of each feature to the predicted suitability class and helps identify the environmental factors most influential in determining solar potential.

All preprocessing, scoring, clustering, and classification procedures were implemented using a Jupyter Notebook–based Python workflow. The complete analysis code and notebooks are publicly available in the Mendeley Data repository (DOI: 10.17632/hkcyvhvmwp.5) to ensure reproducibility.

### 4.4 Highlighting the dataset’s Value

**Data Description:**

Table 2 summarizes Geographic Coverage: The dataset covers latitudes from 10.5° to 36.5° and longitudes from 68.5° to 94.5° (371 locations).

**Table 2:** Summary statistics of key meteorological and geospatial parameters.

Statistic	LAT (°)	LON (°)	GHI (kWh/m²)	CLEAR_GHI (kWh/m²)	ALBEDO	CLOUD_ COVER (%)	TEMP_ 2M (°C)	DEW_ POINT (°C)	PRECIPITATION (mm)
Count	3510	3510	3510	3510	3510	3510	3510	3510	3510
Mean	24.20	79.58	5.01	6.10	0.15	50.67	21.33	11.57	1219.53
Std Dev	6.29	6.43	0.40	0.40	0.06	11.74	10.55	10.36	1089.65
Min	10.50	68.50	3.54	5.22	0.03	21.31	-13.49	-18.86	40.32
25%ile	20.50	74.50	4.73	5.85	0.13	42.91	23.85	10.13	605.53
Median	24.50	78.50	5.02	6.07	0.14	50.76	25.68	13.80	1009.54
75%ile	27.50	82.50	5.30	6.33	0.16	58.52	26.88	18.10	1451.88
Max	36.50	94.50	6.08	7.19	0.44	84.60	29.30	24.59	10375.61

Solar Parameters: Mean GHI (4.98 kWh/m²) and Clear Sky GHI (6.11 kWh/m²) show good solar potential Albedo values (mean 0.15) suggest moderate surface reflectivity

Weather Conditions: Cloud cover varies widely (26–79%), with a median of 52.4%, Temperatures range from -12.9°C to 28.2°C (mean 21.8°C)

Precipitation: Extremely wide range (65–5008 mm), indicating diverse climatic zones Median precipitation (1158 mm) suggests many locations receive substantial rainfall

Variable distribution: As per Fig. 5 the histograms with the overlaid KDEs for GHI, Clear-Sky GHI, albedo, cloud cover, temperature, dew point, and precipitation are illustrated. The central and northwestern zones’ higher irradiance and lower cloud cover are pointed out in contrast to the greater variability in eastern/southern regions.

To investigate the spatial variability and statistical nature of the main environmental parameters, the kernel density estimate (KDE) technique was employed to produce histograms for all seven variables included in the dataset, as illustrated in Fig. 5. The Global Horizontal Irradiance (GHI) distribution shown in Fig. 5 (a) reveals that most of India is well supplied with solar radiation, but the northwest and central zones are the most radiant with their zones already recognized for more solar energy. The Clear-Sky GHI shown in Fig. 5(b) indicative of perfect solar irradiance under clear-sky conditions, continuously surpasses all-sky values and acts as a theoretical upper limit for radiation potential in each region. The surface albedo in Fig. 5 (c) is mostly moderate to low across the majority of areas signifying less reflectivity losses and thus, enabling better photovoltaic performance across a wide range of terrain types.

The cloud cover distribution, illustrated in Fig. 5(d) exhibits sharp regional differences, where the western and central areas have a less dense cloud coverage, and the eastern and southern areas show the opposite with a relatively more varied condition—this all being together with solar energy availability indirectly influenced. The temperature at 2 meters, depicted in Fig. 5(e) is usually warm during the dataset period, has a narrow range distribution which supports effective solar energy output and minimizes the performance deterioration caused by overheating. The dew point variability in Fig. 5(f) is an indicator of the different humidity profiles across the subcontinent which may actually affect condensation risks and the thermal management of photovoltaic systems. To summarize, the precipitation distribution in Fig. 5(g) is the parameter with the widest variability among the others, with heavy rains during the monsoon season in the eastern and southern regions being the main cause of possible impacts on solar panel maintenance, uptime, efficiency, and schedules.

**Fig. 6:**
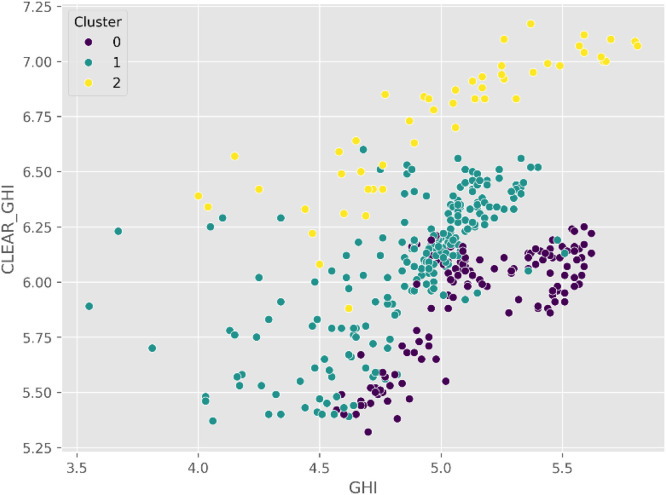
K-means clustering of GHI vs. clear-sky GHI.

Cluster Scatterplot: The visualization represented in Fig. 6 corresponds to the two-dimensional view of the energy received by a surface perpendicular to the sun (Global Horizontal Irradiance, GHI) against the GHI under completely clear sky conditions, with the points colored according to the membership of the respective K-means cluster. There are three clusters that depict the low, moderate, and high irradiance regimes, thus drawing attention to the sites with lower and higher solar potential being separated. Although several suitability categories are defined in later phases, the K-means step was purposely restricted to three clusters to seize the most significant irradiance-driven patterns, after which the final suitability classes were secured by the subsequent scoring and classification.

**Fig. 7:**
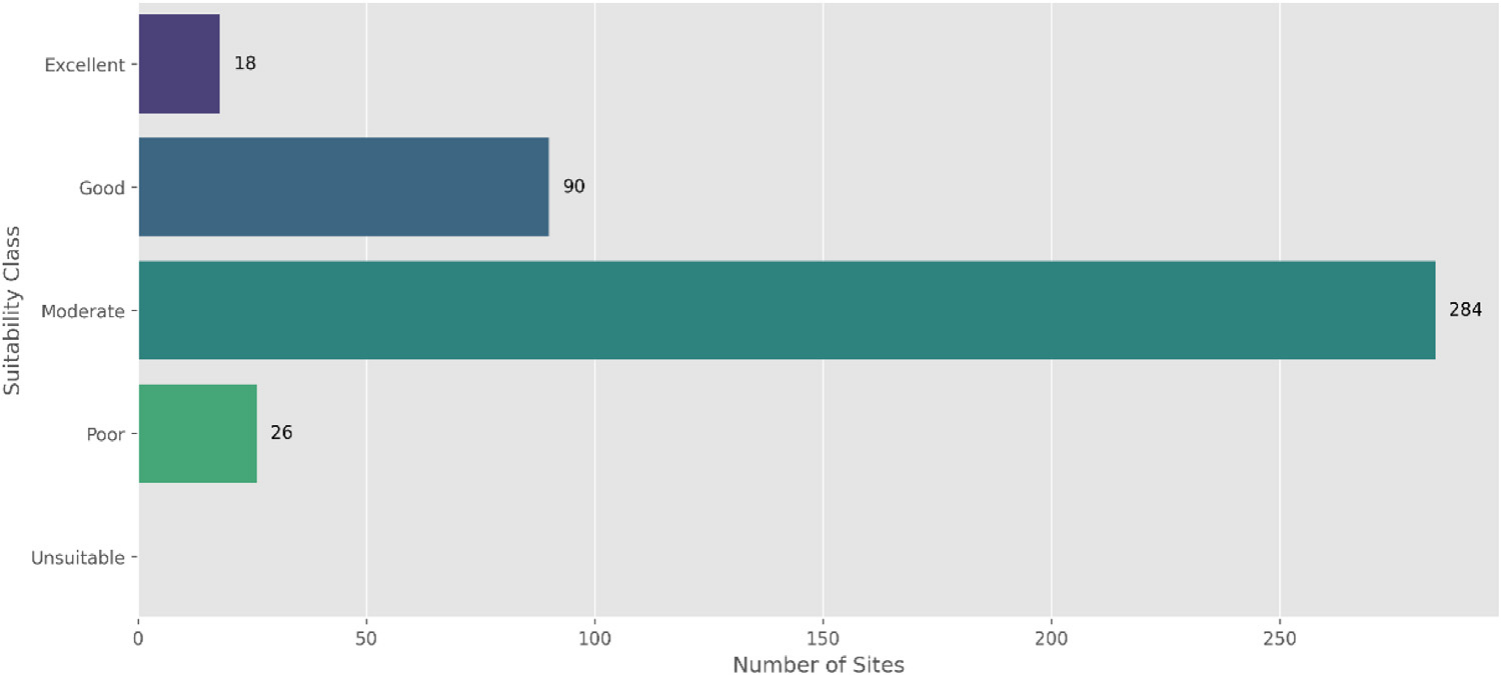
Site distribution by suitability class.

Suitability Class Counts: In accordance with Fig. 7, the horizontal bar chart depicting the site counts for each category is presented. The distribution indicates that 245 locations are predominantly ranked as Moderate while only a few have received Excellent scores.

**Fig. 8:**
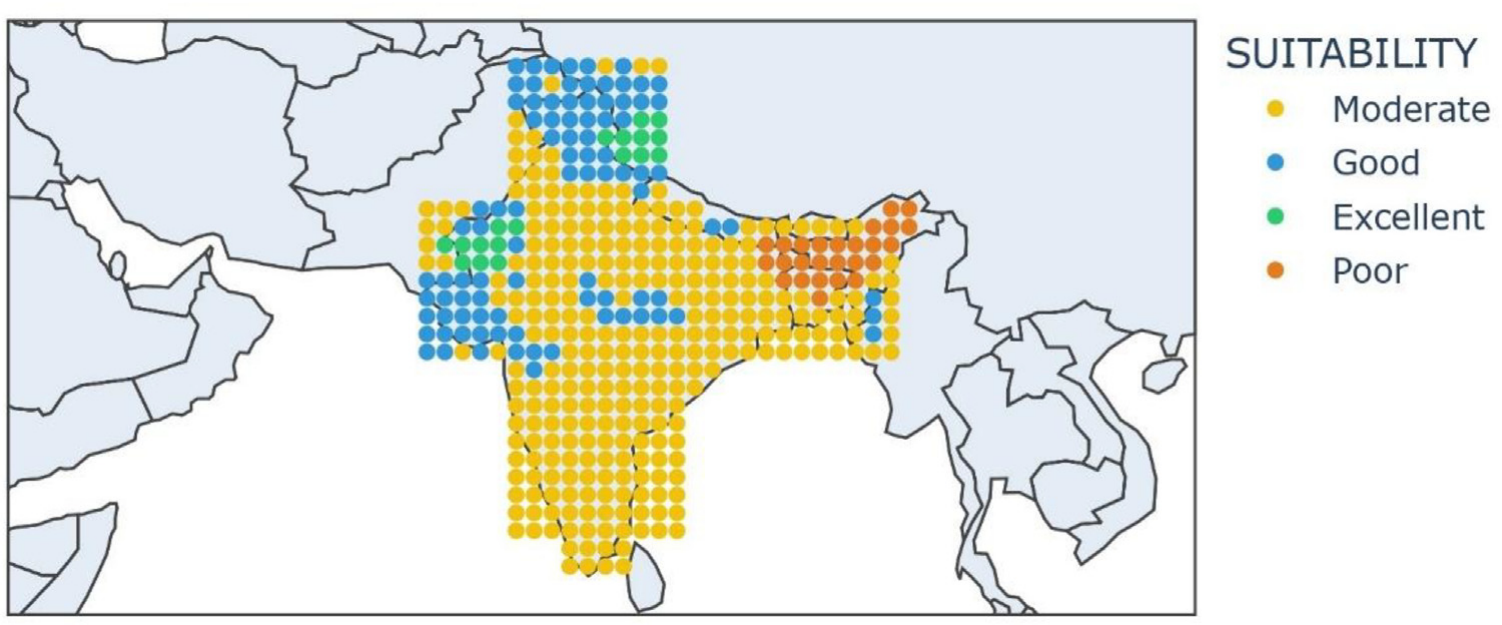
Spatial distribution of solar site suitability: each dot is one of the 371 sites, colored by suitability_class. High-suitability regions (Excellent/Good classes) include Rajasthan and Gujarat, as seen on the map.

Geospatial Suitability Map: As per Fig. 8, A choropleth-style dot map of India, displaying site classes (Excellent, Good, Moderate, Poor) in different colors. It shows the areas with the best suitability in Rajasthan, Gujarat, and central plains, while the northeastern and coastal areas have the lowest scores.

The classification of the sites is given in Fig. 8 along with their respective advisory levels, the high-suitability areas are being pointed out and the low-scoring ones, such as the northeastern and coastal belts, are being highlighted- the reason for the latter being the higher cloud cover and rainfall.

## Limitations

The data’s spatial and temporal resolution is the major restriction of this study. NASA’s POWER dataset comes with global coverage but is restricted to a wide 1 × 1 grid [13], which can overlook the local microclimatic variations that are essential for correct site selection.

Future work could incorporate higher-resolution downscaled data [13] as computational resources allow.

## Ethics Statement

The authors have read and followed the ethical requirements for publication in Data in Brief and confirm that the current work does not involve human subjects, animal experiments, or any data collected from social media platforms.

## CRediT authorship contribution statement

**Yash Shivgan:** Conceptualization, Data curation, Methodology, Writing – original draft. **Chandan Bhirud:** Resources, Validation, Formal analysis. **Anshul Jamgade:** Visualization, Investigation, Software. **Atharva Kulkarni:** Data curation, Writing – review & editing. **Rutuja Patil:** Supervision, Validation. **Gagandeep Kaur:** Supervision, Validation. **David Bassir:** Writing – review & editing, Supervision, Validation. **Ghanshyam G. Tejani:** Writing – review & editing, Supervision, Validation.

## Data Availability

Mendeley DataSaurKshetra: A Dataset for Solar Farms Potential Site Mapping using Suitability parameters in India (Original data).

## Appendix Supplementary Materials

Image, application 1
